# Mesenchymal stem cells in multiple sclerosis - translation to clinical trials

**Published:** 2015

**Authors:** A Dulamea

**Affiliations:** * “Carol Davila” University of Medicine and Pharmacy, Bucharest, Romania; “Fundeni” Clinical Institute, Bucharest, Romania

**Keywords:** mesenchymal stem cells, multiple sclerosis, immunomodulation, immunosuppression, clinical trial

## Abstract

Multiple sclerosis is a chronic inflammatory disease of the central nervous system, characterized by an aberrant activation of the immune system and combining demyelination with neurodegeneration. Studies on experimental models of multiple sclerosis revealed immunomodulatory and immunosuppressive properties of mesenchymal stem cells. Clinical trials using mesenchymal stem cells therapy in multiple sclerosis patients showed tolerability, safety on short term, some immunomodulatory properties reducing the Th1 proinflammatory response and the inflammatory MRI parameters. The author reviews the data about experimental studies and clinical trials using mesenchymal stem cells for the treatment of multiple sclerosis.

**Abbreviations:** MS = multiple sclerosis, RRMS = relapsing-remitting multiple sclerosis, PPMS = primary progressive multiple sclerosis, EAE = experimental autoimmune encephalomyelitis, MOG = myelin oligodendrocyte glycoprotein, PLP = proteolipid protein, MSCs = mesenchymal stem cells, CNS = central nervous system, IT = intrathecal, ALS = amyotrophic lateral sclerosis, MSC-NPs = mesenchymal stem cell-neural progenitors, EDSS = expanded disability status score.

## Introduction

Multiple sclerosis (MS) is a chronic inflammatory disease of the central nervous system (CNS) leading in the end to demyelination and axonal loss. The disease is complex and multifactorial, but it seems that the main etiopathogenic event is represented by an aberrant response of the immune system cells (T and B-lymphocytes) to myelin proteins. There are three forms of the disease evolution. Approximately 80% of the patients have a relapsing-remitting form (RRMS), and two thirds of them will develop a secondary progressive form after 10-15 years from the disease onset. Approximately 20% of the patients develop a progressive form right from the onset, the primary progressive multiple sclerosis (PPMS).

**Experimental studies with mesenchymal stem cells**

Findings about the fact that MSCs inhibit the proliferation of T cell both in vitro [**1**] and in vivo [**2**] suggested that MSCs might be effective in experimental autoimmune encephalomyelitis (EAE), a model for multiple sclerosis, stopping the (auto) immune attack against myelin antigens and promoting nervous tissue repair by their integration into the CNS. In 2005, Zappia et al [**3**] demonstrated that the intravenous injection of syngeneic MSCs improves the clinical course of the experimental autoimmune encephalomyelitis induced by myelin oligodendrocyte glycoprotein (MOG) and reduces demyelination and leukocyte infiltration in the CNS. Cells have these effects only when injected at disease onset or peak; the administration during the chronic stage has no effect. A decisive finding was that T lymphocytes isolated from treated mice did not proliferate in vitro after the exposure to new MOG, an observation suggesting that MSCs induce peripheral immune tolerance. In a subsequent publication, following the intravenous of xenogeneic MSCs (human) in mice with EAE, Zhang and his team found a clinical improvement of the histological scores and some evidence of oligodendrogenesis, possibly through the secretion of neurotrophic factors [**4**]. In 2006, another group confirmed that the administration of MSCs protects neurons from the inflammatory attacks associated with experimental autoimmune encephalomyelitis. They also found a low degree of co-localization (less than 10%) of the labeled human MSCs with cells expressing neural markers [**5**]. Therefore, their findings suggested that a degree of transdifferentiation was present. A subsequent report proved that intravenous injection of MSCs in SJL mice with EAE inhibits the pathogenic humoral immune response by reducing the production of proteolipid protein (PLP)-specific antibodies [**6**]. Moreover, the exposure of PLP-specific encephalitogenic T cells to MSC in vitro prevents the passive transfer of EAE. Some labeled MSCs were detected in the inflamed CNS, close to the infiltrated cells, but no evidence of neural transdifferentiation was found. It is worth noting that neural [**7**] stem cells and, more recently, neural precursor cells derived from human embryonic stem cells [**8**], showed a remarkable benefit following their administration to mice with EAE through the assistance mechanism (“bystander mechanism”) that leads to the immunomodulation of autoreactivity and to neuroprotection [**9**]. Besides the aforementioned immunomodulatory characteristics, MSCs are endowed with many other therapeutic properties, as demonstrated by various studies.

Despite some concerns about their transdifferentiation potential, it is accepted that MSCs can differentiate into mature mesoderm cells such as bone, cartilage and fat [**10**]. In addition, some research groups reported that MSCs showed a minimum potential for transdifferentiation into neurons when transplanted into the CNS of affected mice [**11**]. More importantly, MSCs could promote endogenous repair by recruiting local neural precursor cells, thus leading to a degree of neurogenesis and remyelination [**12**]. Many other features are likely to be relevant to the use of MSCs in multiple sclerosis or in other diseases of the CNS. MSCs showed a strong antioxidant effect in mice affected by EAE [**13**]. The neuroprotective effect may result from the release of anti-apoptotic [**14**] molecules and of neurotrophins [**15**]. These results support the idea that MSCs promote CNS repair by acting both as tolerogenic cells and as bioactive providers of trophic and anti-apoptotic factors which lead to neuroprotection [**16**].

**Clinical trials using mesenchymal stem cells for treatment of multiple sclerosis**

The rationale for using adult stem cells in multiple sclerosis originates in the hope that they might repair the CNS by tissue integration and possibly by differentiating into neural cells. Recent evidence from preclinical studies suggested that MSCs, a sub-class of adult progenitor cells isolated from almost any mesoderm tissue and already clinically studied in various diseases, are an effective cell therapy for EAE. In EAE, intravenous injection of MSCs improves the clinical evolution of the disease and reduces demyelination, immune infiltrates and axonal loss. Surprisingly, these effects do not require grafting of MSCs in the CNS, but are based on the MSCs’ ability to inhibit the pathological response of T and B cells and on the secretion of neuroprotective and pro-oligodendrogenic molecules, facilitating tissue protection and repair. These results led to the conclusion that the administration of MSCs should focus on individuals with ongoing inflammation and before they develop irreversible disability [**17**].

The first small clinical trials in multiple sclerosis have already proven MSCs can be safely used, creating the possibility to move from the clinical use to phase II - extensive studies which address the biological effects of MSCs on markers of disease activity [**18**]. International experts in multiple sclerosis and stem cells, together with immunologists formed the “International Stem Cells Transplantation Study Group” (IMSCTSG) in order to achieve a consensus protocol on the use of MSCs for the treatment of multiple sclerosis, with protocols for cell cultivation and patient treatment. This international consensus treatment protocol based on the efforts of Dr. Uccelli (University of Genova) and Dr. Freedman (University of Ottawa) was conducted and published in 2010 [**19**].

In the consensus document, the authors designed a summary of a clinical trial phase I/ II, to be fully adopted by scientists and clinicians who belong to the MSCT Study Group and which was presented to each national regulator and funding agency as a single-nation research study. This study was designed to evaluate the safety of MSC therapy for patients with relapsing-remitting or secondary progressive multiple sclerosis who do not respond to at least one year of treatment with conventional immunomodulatory therapy, as well as for patients with primary progressive multiple sclerosis.

Autologous MSCs derived from the bone marrow of patients with multiple sclerosis exhibit the same properties as MSCs from healthy donors, in terms of proliferation, phenotype, and differentiation in vivo and immunosuppressive ability [**20**]. A small number of multiple sclerosis patients, who obtained benefits from receiving intravenous or intrathecal infusions of MSCs, was reported. The results of a Phase I/ II study with MSCs used in neurological diseases reported the administration of autologous MSCs in 19 patients with amyotrophic lateral sclerosis (ALS) and 15 patients with multiple sclerosis [**18**]. MSCs were administered as a combination of intravenous and intrathecal injections, at doses of up to 60-70x 10^6 cells/ injection/ patient and the patients monitored for 6 to 28 months, mainly to determine the feasibility and safety of the procedure. None of the patients experienced any significant side effect, except for a mild meningeal irritation such as a headache and fever in those intrathecally injected with MSCs. No infection associated with the (early or late) injection was reported.

Additional data available on the intrathecal use of multipotent mesenchymal stromal cells in multiple sclerosis include a study on 10 patients with progressive multiple sclerosis in Iran [**21**] and a report on 7 patients intrathecally treated in Lebanon [**22**]. They did not communicate significant side effects except for those pertaining to the route of injection (different types of headaches and one case of iatrogenic meningitis) and one case of transient encephalopathy in a patient who intrathecally received an increased dose (100x10^6).

A phase II open-label clinical trial with MSC in secondary progressive multiple sclerosis (SPMS) which was completed and whose results were published (MSCIMS) (ClinicalTrials.gov (NCT00395200) [**23**] included 10 patients with a 14.4 years average duration of disease progression, who received an intravenous dose of 1-2x10^6 cells/ b.w. The patients were clinically monitored for side effects for 4 hours after receiving the dose and also at 3 and 6 months after their treatment. The immediate adverse effects recorded were type I hypersensitivity (pruritus, rash, fever) and were reported in 10% of the subjects after intravenous administration. A statistically significant improvement of the visual acuity and visual evoked potentials was found, as well as an increased optic nerve area following MSCs therapy. There were no identifiable effects on color vision, visual field, macular volume, retinal nerve fiber layer thickness or optic nerve magnetization transfer ratio. The authors’ conclusion was that MSCs were proven safe for use in patients with secondary progressive multiple sclerosis. The study provided evidence of structural, functional and physiological improvement with respect to several goals established for the visual function, which is indicative of the neuroprotective effect of MSCs.

A phase I open label clinical trial of intrathecal (IT) presented a preliminary result at ACTRIMS 2014 [**24**]. Investigators used the administration of autologous mesenchymal stem cell-neural progenitors (MSC-NPs), included 20 patients with progressive MS with established disability (average EDSS=6.0). MSC-NPs were administered IT in three doses of up to 10 million cells per injection, spaced three months apart. Preliminary safety outcomes in the first five study subjects indicated safety and tolerability of the treatment.

Bonab et al (2012) [**25**] conducted an open-label study including 25 patients with progressive MS (EDSS from 4.0 to 6.5) who received a single intrathecal injection of autologous MSCs and were followed-up for 12 months. Associated short-term adverse events of injection consisted of transient low-grade fever, nausea/ vomiting, weakness in the lower limbs and headache. No major delayed adverse effect was reported. 3 patients left the study for personal reasons. The mean (SD) expanded disability status scale (EDSS) score of 22 patients changed from 6.1 (0.6) to 6.3 (0.4). In the MRI evaluation, 15 patients showed no change, whereas 6 patients showed new T2 or gadolinium enhanced lesions (1 lost to follow-up). It seems that MSC therapy can improve/ stabilize the course of the disease in progressive MS in the first year after injection, with no serious adverse effects.

A recent randomized placebo-controlled phase II trial [**26**] included 9 patients who received a dose of 1-2x10^6 MSCs/ Kg body weight intravenously infused. Immunological changes that were consistent with a lower proinflammatory T cell profile, resulting from the decrease in the proportion of IFN-c and with lesser intensity of IL-17-producing CD4+ T cells, and a reduced Th1/ Th17 ratio, were observed. From the MRI point of view, there was a significant trend to lower the mean number of GEL in the second period, suggesting a potential carryover effect of MSCs administration. There were no AE reports after the completion of the 12 months protocol.

A systematic review and meta-analysis of the prospective clinical trials that used intravascular delivery of MSCs (intravenously or intra-arterially) in adult populations or mixed adult and pediatric populations (SafeCell) included 36 studies with a total of 1012 participants with clinical conditions of ischemic stroke, Crohn’s disease, cardiomyopathy, myocardial infarction, graft versus host disease, and healthy volunteers. The meta-analysis of the randomized clinical trials did not detect an association between acute infusion toxicity, organ system complications, infection, death or malignancy. There was a significant association between MSCs and transient fever. However, the results from the experimental studies raised some concerns regarding the tumorigenic potential.

## Conclusions

Mesenchymal stem cells showed effects on innate and adaptive immunity in studies on animal models of multiple sclerosis. Most of the reported trials were uncontrolled open-label phase I studies and included secondary progressive MS, progressive MS, relapsing-remitting and secondary progressive MS and active but unspecific patients, a phase II trial, on secondary progressive MS which included visual and neurophysiological parameters of efficacy and one randomized controlled trial on a small number of relapsing-remitting MS patients not responding to at least one year of approved therapy. The way of administration was intrathecal or intravenous, the number of cells administered and also the number of administrations varied between trials. The patients included were with important disabilities and were followed up to 28 months. All trials reported safety and tolerability of MSCs and stabilization/ mild improvement of the disease. There are still issues to be clarified about the migration potential and homing into the central nervous system of MSCs and also about the possibility of tracking the distribution of MSCs in humans. The small number of patients included in these trials and the differences between the trial designs consist of limitations in the interpretation of data and justify the necessity for further randomized multicentric controlled trials.

**Source of funding:** none

**Disclosures:** None

## References

[1] Di Nicola M (2002). Human bone marrow stromal cells suppress T-lymphocyte proliferation induced by cellular or nonspecific mitogenic stimuli. Blood.

[2] Bartholomew A (2002). Mesenchymal stem cells suppress lymphocyte proliferation in vitro and prolong skin graft survival in vivo. Exp.Hematol.

[3] Zappia E (2005). Mesenchymal stem cells ameliorate experimental autoimmune encephalomyelitis inducing T cell anergy. Blood.

[4] Zhang J (2005). Human bone marrow stromal cell treatment improves neurological functional recovery in EAE mice. Exp.Neurol.

[5] Zhang J (2006). Bone marrow stromal cells reduce axonal loss in experimental autoimmune encephalomyelitis mice. J Neurosci Res.

[6] Gerdoni E (2007). Mesenchymal stem cells effectively modulate pathogenic immune response in experimental autoimmune encephalomyelitis. Ann Neurol.

[7] Martino G, Pluchino S (2006). The therapeutic potential of neural stem cells. Nat Rev Neurosci.

[8] Aharonowiz M (2008). Neuroprotective effect of transplanted human embryonic stem cell-derived neural precursors in an animal model of multiple sclerosis. PLoS One.

[9] Uccelli A, Mancardi G (2010). Stem cell transplantation in multiple sclerosis. Curr Opin Neurol.

[10] Pittenger MF (1999). Multilineage potential of adult human mesenchymal stem cells. Science.

[11] Kopen GC, Prockop DJ, Phinney DG (1999). Marrow stromal cells migrate throughout forebrain and cerebellum, and they differentiate into astrocytes after injection into neonatal mouse brains. Proc.Natl.Acad.Sci U.S.A..

[12] Bai L (2009). Human bone marrow-derived mesenchymal stem cells induce Th2-polarized immune response and promote endogenous repair in animal models of multiple sclerosis. Glia.

[13] Lanza C (2009). Neuroprotective Mesenchymal Stem Cells are endowed with a potent antioxidant effect in vivo. J Neurochem.

[14] Kemp K (2009). Mesenchymal stem cell-secreted superoxide dismutase promotes cerebellar neuronal survival. J Neurochem.

[15] Wilkins A (2009). Human bone marrow-derived mesenchymal stem cells secrete brain-derived neurotrophic factor which promotes neuronal survival in vitro. Stem Cell Res.

[16] Uccelli A, Moretta L, Pistoia V (2008). Mesenchymal stem cells in health and disease. Nat Rev Immunol.

[17] Uccelli A, Laroni A, Freedman MS (2011). Mesenchymal stem cells for the treatment of multiple sclerosis and other neurological diseases. Lancet Neurol.

[18] Karussis D (2010). Safety and immunological effects of mesenchymal stem cell transplantation in patients with multiple sclerosis and amyotrophic lateral sclerosis. Arch Neurol.

[19] Freedman MS (2010). The therapeutic potential of mesenchymal stem cell transplantation as a treatment for multiple sclerosis: consensus report of the International MSCT Study Group. Mult Scler.

[20] Mallam E (2010). Characterization of in vitro expanded bone marrow-derived mesenchymal stem cells from patients with multiple sclerosis. Mult Scler.

[21] Mohyeddin Bonab M (2007). Does mesenchymal stem cell therapy help multiple sclerosis patients?. Report of a pilot study. Iran J Immunol.

[22] Yamout B (2010). Bone marrow mesenchymal stem cell transplantation in patients with multiple sclerosis: a pilot study. J Neuroimmunol.

[23] Connick P, Kolappan M, Patani R, Scott MA (2011). The mesenchymal stem cells in multiple sclerosis (MSCIMS) trial protocol and baseline cohort characteristics: an open-label pre-test: post-test study with blinded outcome assessments. Trials.

[24] Harris VK, Vyshkina T, Chirls S, Sadiq SA (2014). Intrathecal administration of mesenchymal stem cell-neural progenitors in multiple sclerosis: an interim analysis of a phase I clinical trial. abstract ACTRIMS.

[25] Bonab MM, Sahraian MA, Aghsaie A, Karvigh SA, Hosseinian SM, Nikbin B, Lotfi J, Khorramnia S, Motamed MR, Togha M, Harirchian MH, Moghadam NB, Alikhani K, Yadegari S, Jafarian S, Gheini MR (2012). Autologous mesenchymal stem cell therapy in progressive multiple sclerosis: an open label study. Curr Stem Cell Res Ther.

[26] Llufriu S, Sepulveda M, Blanco Y, Marın P, Moreno B (2014). Randomized Placebo-Controlled Phase II Trial of Autologous Mesenchymal Stem Cells in Multiple Sclerosis. PLoS ONE.

